# Melatonin improves stroke through MDM2-mediated ubiquitination of ACSL4

**DOI:** 10.18632/aging.205469

**Published:** 2024-01-29

**Authors:** Qing Ji, Le Zhang, Hui Ye

**Affiliations:** 1 School of Medicine and Health, Anyang Vocational and Technical College, Anyang, Henan 455008, China; 2 Department of Pharmacy, Chongqing Yubei District People’s Hospital, Yubei, Chongqing 401120, China; 3 Department of Neurosurgery, Yulin Hospital, The First Affiliated Hospital of Xi'an Jiaotong University, Yulin, Shaanxi 719000, China

**Keywords:** ischemic stroke, ferroptosis, melatonin, MDM2, MCAO

## Abstract

The objective of this study is to investigate the impact of melatonin on ischemic brain injury and elucidate its underlying molecular mechanism. In this investigation, a mouse model of middle cerebral artery occlusion (MCAO) was established using the thread occlusion method, followed by treatment with two different doses of melatonin: 5 mg/kg and 10 mg/kg. Additionally, HT-22 cells were subjected to oxygen-glucose deprivation/reoxygenation (OGD/R) and treated with varying concentrations of melatonin. The findings demonstrated that melatonin significantly reduced the extent of cerebral ischemia, nerve damage, brain edema, and neuronal apoptosis in MCAO mice. *In vitro* experiments further revealed that melatonin effectively enhanced cell proliferation while reducing cell apoptosis and reactive oxygen species (ROS) production following OGD/R treatment. Mechanistic investigations unveiled that melatonin exerted its protective effect by inhibiting ferroptosis through modulation of MDM2-mediated ubiquitination of ACSL4. In summary, this study suggests that melatonin regulates the MDM2/ACSL4 pathway to safeguard against ischemic brain injury, thereby providing novel therapeutic targets for such conditions.

## INTRODUCTION

Ischemic stroke, a condition with potentially fatal consequences, causes millions of deaths globally annually [1]. In China, stroke has emerged as the leading cause of mortality, resulting in millions of fatalities each year [2]. Especially the elderly are more prone to be affected. However, treatment of ischemic stroke still faces some challenges, including treatment bottlenecks [3]. The elderly population is particularly susceptible to being impacted, with treatment of ischemic stroke encountering certain obstacles and bottlenecks [4]. Furthermore, given the unfavorable outlook for ischemic stroke characterized by elevated rates of disability and mortality, it is imperative to prioritize prevention efforts and timely identification [5]. Hence, the enhancement of molecular mechanism investigation on ischemic stroke and identification of effective therapeutic approaches will significantly contribute to the reduction in both occurrence and fatality rates associated with stroke.

Stroke is a severe medical condition, and ferroptosis plays a pivotal role in its pathogenesis. Subsequent to an episode of ischemic stroke, there is an accumulation of iron within the cerebral neurons and glial cells, leading to cellular damage [6]. Furthermore, the accumulation of iron also triggers the production of reactive oxygen species (ROS), which further worsens cellular structural and functional impairments. Additionally, iron has the ability to initiate apoptotic signaling pathways in both neuronal and glial cells, ultimately resulting in their demise [7]. Simultaneously, iron accumulation also disrupts intracellular proteostasis, thereby exacerbating the extent of cellular damage [8].

However, recent research has indicated that the regulation of stroke and ferroptosis could potentially be influenced by Melatonin (MT) [9]. Melatonin is thought to possess properties that can reduce oxidative stress and inflammatory reactions triggered by stroke, potentially due to its antioxidant and anti-inflammatory effects [10]. Furthermore, the regulation of iron metabolism and transportation processes can be achieved by Melatonin. It has the ability to hinder excessive release and accumulation of iron, ultimately leading to a decrease in cytotoxicity caused by iron [11, 12]. Studies have additionally discovered that the regulation of cell apoptosis and neurotransmitter release pathways by Melatonin can mitigate nerve cell damage resulting from stroke [9]. While additional research is necessary to fully comprehend the role of Melatonin in stroke and its regulation of iron-mediated death, current evidence suggests that Melatonin could potentially offer therapeutic and preventive benefits for stroke. Further investigation will shed light on the precise mechanism by which Melatonin operates during a stroke, thereby providing a theoretical foundation for the development of novel treatment strategies. Exploring the interplay between Melatonin and ferroptosis mechanisms may open up new avenues for addressing stroke.

This research aims to investigate the regulatory function of Melatonin in iron-induced cell death triggered by stroke and its underlying molecular mechanism using techniques from the field of molecular biology. The findings obtained from our study will establish a foundation for potential treatments for ischemic stroke and the design of specific therapeutic approaches.

## MATERIALS AND METHODS

### Animals and middle cerebral artery occlusion (MCAO) model

All C57BL/6 mice, aged between 8 and 10 weeks, were acquired from Beijing Charles River Laboratory Animal Company. They were accommodated in a facility free from pathogens, following a light/dark cycle of 12 hours each, with unrestricted access to food and water. All animal studies were performed in accordance with the Ethical Guidelines for the Use and Care of Laboratory Animals and were approved by the Animal Ethics Committee of the First Affiliated Hospital of Xi’an Jiaotong University.

All mice underwent a 12-hour period of fasting and water deprivation. The MCAO model was induced using the internal carotid artery occlusion technique. Anesthesia was administered via intraperitoneal injection of pentobarbital at a dosage of 60 mg/kg, and the mice were positioned in a prone position. A disinfection procedure was performed on the neck, followed by making a 1.5 cm incision in the middle region. Subsequently, the subcutaneous tissue, glands, and muscles were carefully separated to expose both the right common carotid artery and internal carotid artery.

To establish arterial occlusion, knots were tied at both ends of the external carotid artery. Then, a small incision was made between these knots to allow for insertion of a thread plug into this area. By pulling on the external carotid artery gently, we ensured that the thread plug gradually entered approximately 10 mm into the internal carotid artery; if any resistance was encountered during this process, entry would be halted accordingly. Once properly positioned, we secured the thread plug in place before suturing each layer of tissue sequentially to close up the incision site meticulously. Additionally, disinfection measures were implemented to prevent infection.

After restoring cerebral blood flow successfully through these procedures described above, subsequent experiments could be conducted after an interval of 48 hours had passed since surgery initiation. In contrast to tMCAO group animals experiencing cerebral ischemia due to complete penetration by suture plugs into their arteries (as detailed earlier), those assigned to sham operation group underwent identical surgical steps except that only partial penetration (approximately 5 mm) occurred without inducing cerebral ischemia.

### Oxygen and glucose deprivation/reoxygenation (OGD/R)

For the *in vitro* assays, we utilized the OGD/R assay. HT-22 cells (obtained from Procell Co., Ltd.) were exposed to 1% oxygen in glucose-free DMEM at 37°C for a duration of 4 hours to induce OGD. The medium was allowed to equilibrate to 1% hypoxia within two hours under hypoxic conditions. To ensure that the effects of hypoxia were observed, an adequate amount of sugar-free DMEM was added to the dishes of the hypoxia workstation four hours prior to OGD. Oxygen-glucose deprivation manipulations were performed in a hypoxic workstation during which HT-22 cells were incubated in complete medium and saturated with a humidified atmosphere consisting of 5% CO_2_ and 95% air for eight hours before harvesting. Control cells underwent similar washing and medium changes but remained at a constant temperature of 37°C in complete medium with an atmosphere consisting of 5% CO_2_ and 95% air throughout the experiment. Melatonin was dissolved in absolute ethanol and further diluted into basal medium for final concentrations. In this study, we used MT-L (20 μM) and MT-H (40 Μm), both administered thirty minutes prior to OGD/R treatment followed by treatment as planned.

### TTC assay

Following a 48-hour MCAO operation, carefully separate the tissue at the base of the skull to obtain complete brain tissue. Place this brain tissue onto a mold and proceed with cutting it into four coronal slices, each approximately 2 mm thick. Submerge these sliced brain tissues in TTC solution and incubate them at 37°C for 30 minutes. After incubation, remove the brain slices and fix them with Paraformaldehyde for a duration of 24 hours. Utilize a scanner to scan and image the fixed slices. Normal brain tissues will display red areas, while ischemic brain tissues will appear as white areas. The volume of cerebral infarction can be measured using ImageJ software.

### Cell viability assay

In this research, the cell viability of cells in each treatment group was assessed using the CCK-8 assay. The experimental procedures were conducted following the guidelines provided by the supplier, and the absorbance at 450 nm after treatment was measured for each treatment group.

### Cell transfection

The synthesis of siRNA (si-MDM2) and overexpressed vectors was outsourced to Genepharma company located in Shanghai, China. The HT-22 cells were prepared for transfection 24 hours prior, and the amount of siRNA transfected ranged from 20 to 100 nmol based on cell tolerance and transfection efficiency. For the transfection process, a reaction system consisting of 4 mL serum-free medium was used. After incubation for 8 hours, the medium was replaced with fresh medium and further cultured for an additional 48 hours.

### Real-time fluorescence quantitative PCR (qRT-PCR)

The TRIzol method was employed to extract total RNA from the samples, followed by cDNA reverse transcription using the Transcriptor First Strand cDNA Synthesis Kit (Taraka, Japan). β-Actin served as an internal reference for normalization purposes. Quantitative PCR amplification was carried out using Fast Start Universal SYBR Green Master (Rox). All primer sequences were shown in Supplementary Table 1.

### Western blotting (WB)

The protein was obtained from RIPA lysate and its concentration was determined using the BCA method. Electrophoresis was performed on a polyacrylamide gel. Following electrophoresis, the membrane was transferred to a 0.45 μm PVDF membrane and sealed with 5% skim milk for 2 hours. The primary antibody was incubated overnight at 4°C, followed by washing with TBST (3 times, 10 minutes each). HRP labeled IgG from the same source as the primary antibody (anti-MDM2, ab259265, dilution of 1:1000; anti-ACSL4, ab155282, dilution of 1:10000; and anti-β-actin, ab8226, concentration of 1 μg/ml) were selected and incubated at room temperature for 1.5 hours. The membrane was then washed with TBST (3 times, 10 minutes each), exposed to film for imaging purposes before development and fixing procedures were carried out.

### Hematoxylin and eosin (H&E) and Nissl staining

Brains were fixed overnight in 4% paraformaldehyde, embedded in paraffin, and serially sectioned, deparaffinized, and hydrated. The sections were stained with H&E for routine histological examination with a light microscope.

### ACSL4 ubiquitination assay

To conduct the ubiquitination assay, we introduced the designated plasmid into the cells and subsequently exposed them to 20 μM MG132 for a duration of 6 hours. Following this, we collected the cells using EBC buffer and employed sonication to disrupt their suspension. The resulting suspensions were then subjected to centrifugation at 12,000 × g for a period of 15 minutes, allowing us to collect the supernatant. Subsequently, we incubated the supernatant with sepharose beads conjugated with Flag antibody while gently rotating them at a temperature of 4°C for no less than 12 hours. Afterward, we performed three washes on the samples using EBC buffer. To prepare for further analysis, we mixed the beads with loading buffer (2× concentration) and heated them by boiling for a total of 6 minutes. Finally, these samples underwent western blot analysis utilizing the specified antibody.

### Measurement of lipid ROS

The HT-22 cells were cultured in 96-well plates with glass bottoms, with a seeding density of 3,000 cells per well. The cells were allowed to grow overnight before being subjected to treatment. Following the treatment, the cells were stained simultaneously using BODIPY-C11 dye and DAPI. After washing away any excess dye, digital images of the stained cells were captured using a laser scanning confocal microscope (Olympus) equipped with a 60× objective lens.

### Glutathione measurements

HT-22 cells were cultured in 6-well plates with a seeding density of 5 × 104 cells per well. Following the designated treatments for a duration of 12 hours, cellular samples were collected and processed using an assay kit (manufactured in Nanjing, China) to quantify the overall intracellular glutathione (GSH) levels, following the provided instructions.

### Statistical analysis

The data was analyzed using GraphPad Prism 8.0 software, and the experimental results were presented as mean ± standard deviation (Mean ± SD). One-way analysis of variance was employed to compare multiple groups, while pairwise comparison between groups was conducted using the Tukey test. An independent sample t-test was used for comparing two groups. The observed difference exhibited statistical significance at *P* < 0.05 level.

### Data availability

The datasets used and/or analyzed during the current study are available from the corresponding author on reasonable request.

## RESULTS

### Melatonin improves neuronal damage caused by stroke

**Figure 1:**
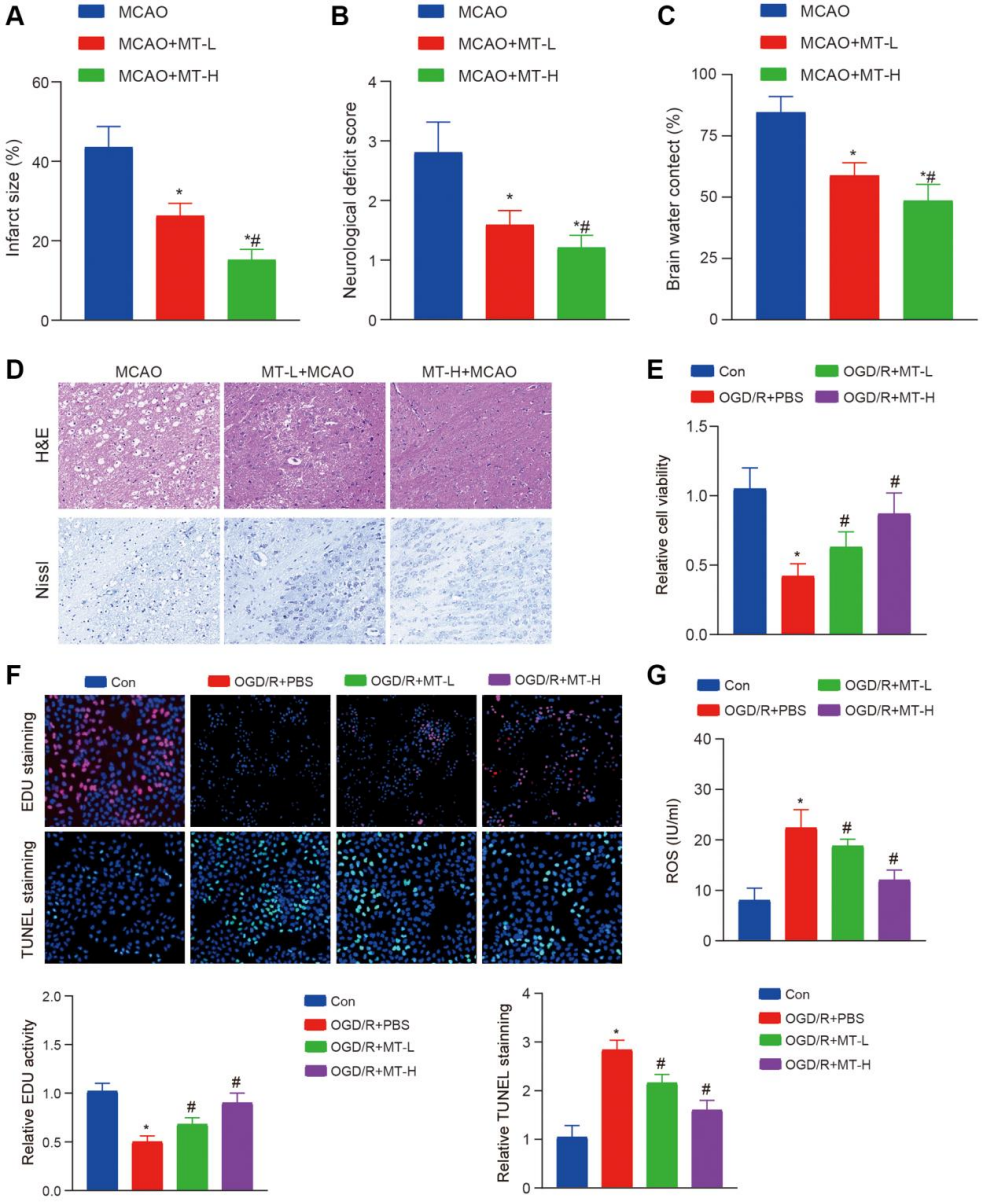
**Melatonin improves brain tissue and neuronal damage caused by stroke.** (**A**) TTC staining reveals that Melatonin at doses of 5 and 10 mg/kg significantly reduces the volume of infarction compared to the MCAO group. (**B**, **C**) The administration of Melatonin at doses of 5 and 10 mg/kg markedly decreases neural damage score and brain water content in comparison to the MCAO group. (**D**) H&E and Nissl staining demonstrate that Melatonin at doses of 5 and 10 mg/kg mitigates neuronal damage when compared to the MCAO group, with a more pronounced effect observed at a dose of 10 mg/kg. (**E**) CCK-8 assay indicates that treatment with Melatonin at concentrations of 0.5 and 1 mM enhances HT-22 cell viability relative to the OGD/R group. (**F**) The EdU and TUNEL assay indicate that treatment with Melatonin at concentrations of 0.5 and 1 mM enhances HT-22 proliferation and apoptosis relative to the OGD/R group. (**G**) DCFH-DA staining shows that exposure to Melatonin at concentrations of 0.5 and 1 mM reduces ROS levels compared to the OGD/R group. The data are presented as mean ± SD. Statistical significance is denoted by ^*^P < 0.05 versus either the Con groups, ^#^*P* < 0.05 versus either the MCAO or OGD/R groups.

To investigate the potential therapeutic effects of Melatonin on brain damage caused by MCAO, we administered two different doses of Melatonin (5 mg/kg and 10 mg/kg) to mice with MCAO-induced injury. Subsequently, we evaluated the extent of post-ischemic damage. The findings revealed a significant reduction in the ischemic area following treatment with both doses of Melatonin (Figure 1A). In addition, when comparing neural damage and cerebral edema, Melatonin demonstrated significant protective effects (Figure 1B, 1C). The H&E and Nissl staining results showed that both doses of Melatonin effectively improved the neuronal damage caused by MCAO. Moreover, the higher dose group exhibited more pronounced effects compared to the lower dose group (Figure 1D).

To further explore the neuroprotective properties of Melatonin, we conducted an OGD/R experiment using HT-22 cells to validate its efficacy. Following treatment, we assessed cell viability and apoptosis and observed a significant improvement in cell proliferation capacity and suppression of apoptosis with both Melatonin doses (Figure 1E, 1F). In addition, we conducted an assessment on oxidative harm and observed a decrease in the generation of ROS following the administration of Melatonin treatment (Figure 1G). These findings indicate that the administration of MT may enhance the recovery of brain tissues and neurons affected by MCAO or OGD/R.

### The role of the ferroptosis in treating MCAO with Melatonin

**Figure 2:**
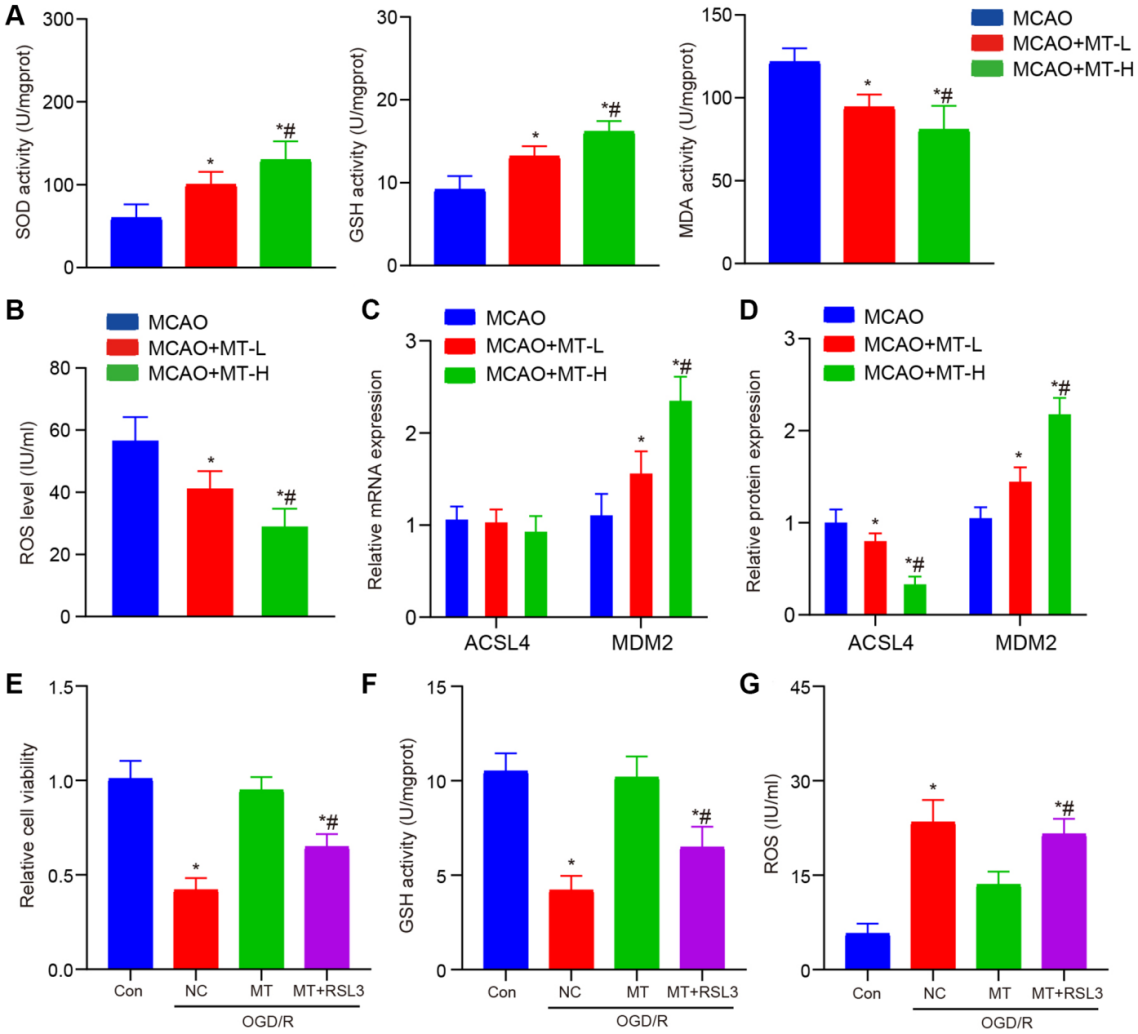
**Melatonin improves brain tissue and neuronal damage caused by stroke.** (**A**) In brain tissues, melatonin administration at doses of 5 and 10 mg/kg resulted in increased levels of SOD and GSH, while decreasing the level of MDA compared to the MCAO group. (**B**) Melatonin treatment at doses of 5 and 10 mg/kg reduced ROS accumulation in brain tissues when compared to the MCAO group. (**C**) The ACSL4 and MDM2 mRNA levels, as determined by qRT-PCR analysis, were compared between the groups treated with melatonin (at doses of 5 and 10 mg/kg) and the MCAO group. (**D**) The ACSL4 and MDM2 protein levels, as determined by WB analysis, were compared between the groups treated with melatonin (at doses of 5 and 10 mg/kg) and the MCAO group. (**E**) The protective effects of melatonin (at a dose of 10 mg/kg) on HT-22 cell viability were reversed by RSL3, as demonstrated by CCK-8 assay results. (**F**, **G**) The inhibitory effects of melatonin (at a dose of 10 mg/kg) on ROS and MDA levels in HT-22 cells were abolished by RSL3 according to ELISA assay findings. Data are presented as mean ± SD. ^*^P < 0.05 vs. Con group; ^#^P < 0.05 vs. MT treatment group.

According to recent studies, the disease process may be influenced by melatonin through its regulation of the ferroptosis pathway [13–15]. Hence, our initial focus was on identifying the presence of oxidative harm in MCAO tissues that were subjected to melatonin treatment. Remarkably, we observed a substantial enhancement in the levels of SOD and GSH, along with a suppression in the expression of MDA upon administering two different doses of melatonin (Figure 2A). In addition, we observed the levels of ROS and discovered their potential to impede the accumulation of ROS induced by MCAO (Figure 2B). Finally, the expression of ACSL4 was detected and it was observed that melatonin exhibited inhibitory effects on the protein expression of ACSL4 without significantly affecting its mRNA expression (Figure 2C, 2D).

To further investigate the impact of melatonin on MCAO and its regulation of ferroptosis, we administered high doses of both melatonin and RSL3 simultaneously to the HT-22 OGD/R model. Our findings revealed that co-treatment with RSL3 effectively counteracted the enhanced cell proliferation ability induced by melatonin (Figure 2E). At the simultaneous administration, the collective therapy notably counteracted the positive impact of melatonin on oxidative harm (Figure 2F, 2G). In summary, the findings of this study indicate that melatonin exerts a neuroprotective effect in cases of cerebral ischemia through its regulation of the iron death pathway. Melatonin effectively reduces lipid peroxidation and ROS production caused by iron, while also inhibiting ferroptosis induced by iron through the modulation of ACSL4 protein expression. Targeting the regulation of the iron death pathway, which is influenced by melatonin, could potentially serve as a novel therapeutic approach for treating cerebral ischemia.

### MDM2 regulates the ferroptosis process by regulating ACSL4 ubiquitination

**Figure 3:**
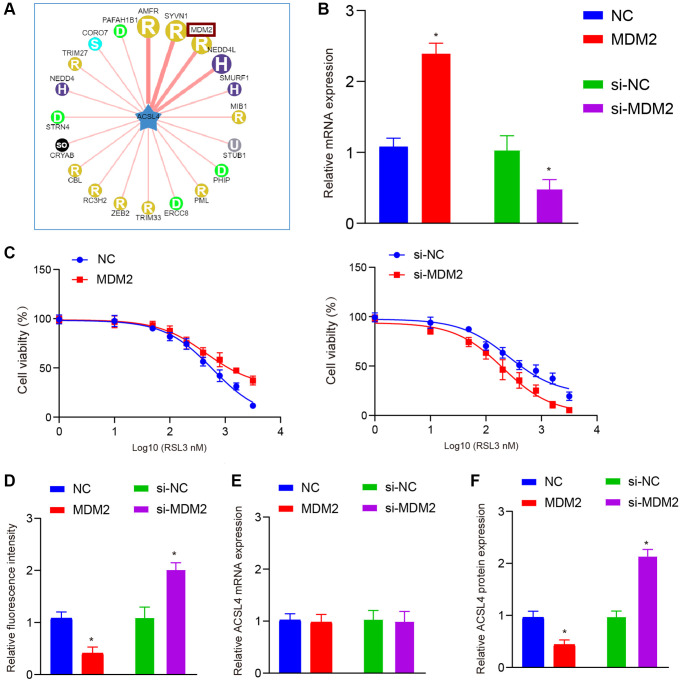
**MDM2 affects ferroptosis by regulating ACSL4 protein levels, not mRNA levels.** (**A**) Ubibrowser database is used to predict the ubiquitin E3 ligases that ACSL4 may interact with. (**B**) QRT-PCR shows overexpression and knockdown efficiency of MDM2 in HT-22 cells. (**C**) CCK-8 assay shows MDM2 overexpression increased while MDM2 knockdown decreased the IC50 of RSL3 in HT-22 cells. (**D**) DCFH-DA staining shows MDM2 overexpression inhibited while MDM2 knockdown increased ROS levels in HT-22 cells treated with RSL3. (**E**) QRT-PCR shows MDM2 overexpression or knockdown did not affect ACSL4 mRNA levels. (**F**) Western blot shows MDM2 overexpression decreased while MDM2 knockdown increased ACSL4 protein levels. Data are presented as mean ± SD. ^*^P < 0.05 vs. NC or si-NC group.

Our data demonstrate that melatonin specifically modulates the horizontal expression of ACSL4 protein, implying a potential involvement of post-translational modifications. Consequently, we employed the Ubibromser database to predict plausible E3 ligases that could interact with ACSL4, leading us to identify three highly correlated E3 ligases (AMFR, SYVN1, and MDM2) (Figure 3A). Subsequent literature screening revealed that MDM2 exerts inhibitory effects on ferroptosis [16, 17]. Therefore, we selected MDM2 as our target molecule for further validation. Hence, we initially assessed the impact of melatonin treatment on MDM2 expression and observed that two different doses of melatonin were able to considerably enhance the expression levels of MDM2 (Figure 2C, 2D). Furthermore, to verify the effect of MDM2 expression on ferroptosis, we constructed overexpression and knockdown models (Figure 3B). Our results showed that overexpression of MDM2 could significantly increase the IC50 of RSL3, while inhibiting the expression of MDM2 significantly decreased the IC50 (Figure 3C). Then we found that overexpression of MDM2 could significantly inhibit ROS levels, while the si-MDM2 group had the opposite effect (Figure 3D). To confirm our hypothesis, we assessed the mRNA and protein levels of ACSL4 and observed that modulation of MDM2 expression did not significantly impact ACSL4 mRNA levels. However, it exhibited a negative regulatory effect on ACSL4 protein abundance (Figure 3E, 3F).

**Figure 4:**
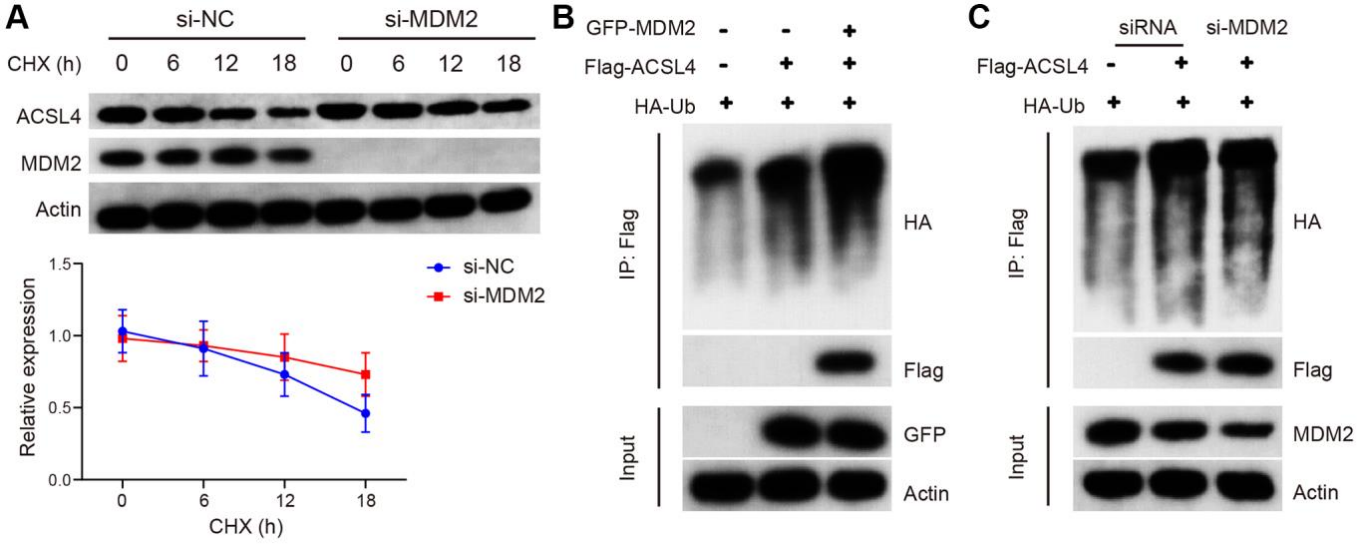
**MDM2 regulates the ubiquitination of ACSL4 protein.** (**A**) The half-life of ACSL4 protein in CHX chase assay was found to be increased by si-MDM2, as shown by WB analysis. (**B**) Overexpression of MDM2 resulted in an increase in polyubiquitinated ACSL4 in HT-22 cells, as observed through IP-WB analysis. (**C**) Knockdown of MDM2 led to a decrease in polyubiquitinated ACSL4 in HT-22 cells, as demonstrated by IP-WB analysis. Data are presented as mean ± SD. ^*^P < 0.05 vs. NC or si-NC group.

The translation of ACSL4 protein is regulated by MDM2, as indicated by these findings. To investigate the impact of MDM2 on the stability of ACSL4 protein, we treated cells inhibited by MDM2 with chalcone (CHX), a selective inhibitor of protein synthesis at specific time intervals. Western blot analysis revealed that si-MDM2 increased the lifespan of ACSL4. These results suggest that MDM2 reduces the stability of ACSL4 protein through an elevation in its ubiquitination level (Figure 4A). Next, we examined the impact of MDM2 on the ubiquitination process of ACSL4. As anticipated, our findings revealed that the upregulation of MDM2 led to an increase in polyubiquitin-linked ACSL4, whereas the downregulation of MDM2 resulted in a decrease in the level of polyubiquitinated ACSL4 (Figure 4B, 4C). These results indicate that MDM2 promotes ACSL4 degradation by increasing its ubiquitination.

### MDM2 mediated melatonin treatment effect

**Figure 5:**
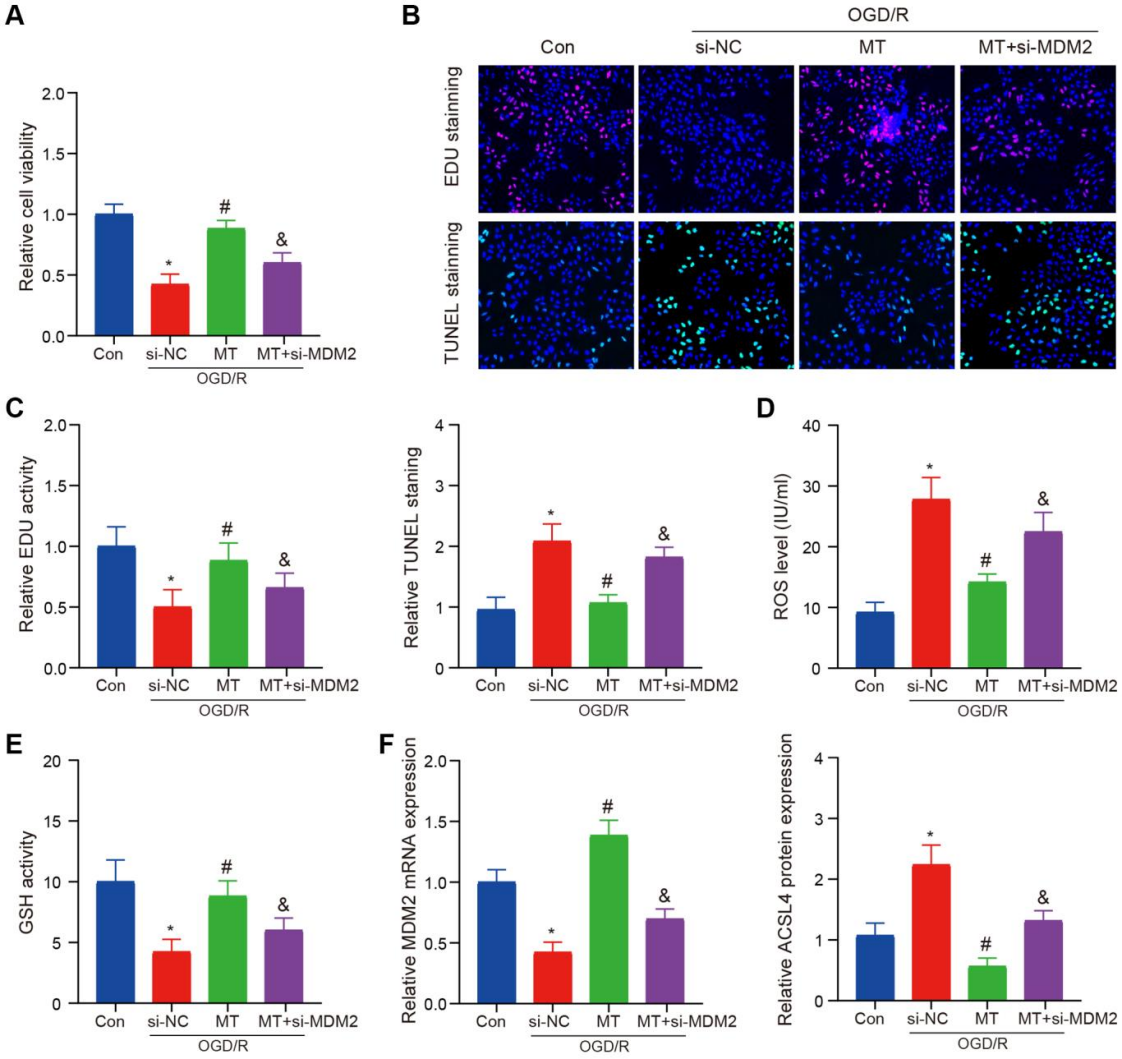
**MCAO model verifies the role of MDM2 in regulating Melatonin to improve brain injury.** (**A**) The CCK-8 assay indicates that the protective effects of Melatonin on HT-22 cell viability were reversed upon MDM2 knockdown. (**B**, **C**) The EdU and TUNEL assay demonstrates that MDM2 knockdown eliminates the effects of Melatonin in improving HT-22 cell proliferation and apoptosis. (**D**) DCFH-DA staining shows that MDM2 knockdown hinders the effects of Melatonin in reducing ROS levels. (**E**) Detection of GSH reveals that MDM2 knockdown nullifies the effects of Melatonin in increasing GSH levels. (**F**) QRT-PCR and WB analysis illustrates that MDM2 knockdown reverses the inhibitory effects of Melatonin on ACSL4 expression. Data are presented as mean ± SD. ^*^P < 0.05 vs. Con group; ^#^*P* < 0.05 vs. si-NC group; ^&^*P* < 0.05 vs. MT-H group.

We utilized siRNA to establish an MDM2 inhibition model for validating the neuroprotective effect of melatonin against OGD/R in HT-22 cells. Our findings demonstrated that the protective impact of melatonin on neuronal injury was abrogated upon suppression of MDM2 expression, as evidenced by the reversal of cell proliferation and apoptosis inhibition (Figure 5A–5C). We observed a reversal of this phenomenon, even when administering a high dosage of Melatonin. Subsequently, we assessed the levels of ROS and GSH and noted that the beneficial impact of Melatonin diminished upon inhibition of MDM2 expression (Figure 5D, 5E). Finally, we also validated the expression of MDM2 and ACSL4, which supported our previous hypothesis (Figure 5F).

## DISCUSSION

Melatonin, a hormone naturally produced in the body mainly by the pineal gland, plays a crucial role in regulating circadian rhythms and sleep patterns. Recent studies have highlighted its potential therapeutic benefits due to its antioxidant, anti-inflammatory, and anti-apoptotic properties. As a result, it has gained significant attention in research related to various diseases such as neurodegenerative disorders, cardiovascular conditions, and tumors. This investigation utilized mouse models of MCAO and HT-22 cells subjected to OGD/R to demonstrate that Melatonin can effectively mitigate neuronal damage caused by ischemic stroke. Furthermore, an exploration into its mechanism revealed the involvement of ferroptosis and MDM2 as important factors.

Ferroptosis is a recently identified form of programmed cell death triggered by the excessive accumulation of iron ions [18]. Ferroptosis also plays a crucial role in various diseases, including neurodegenerative diseases, cardiovascular diseases, and tumors. ACSL4 is a critical determinant of ferroptosis, executing ferroptosis by catalyzing lipid biosynthesis [19, 20]. The findings of this study suggest that melatonin can enhance the ferroptosis level in neuronal cells by inhibiting ACSL4 expression. Interestingly, no significant difference was observed in the regulation of ACSL4 mRNA, indicating a potential role for post-translational modification. Protein ubiquitination is a process where intracellular proteins are classified and modified specifically through a series of enzymes including ubiquitin-activating enzymes, conjugating enzymes, ligases and degrading enzymes. This process plays an important role in protein localization, metabolism, function, regulation and degradation as well as almost all life activities such as cell cycle progression, proliferation, apoptosis, differentiation, metastasis and gene expression [21–24]. Furthermore, it is involved in transcriptional regulation signal transduction damage repair inflammation and immunity. Ubiquitin has been implicated in diseases such as cancer and nervous system disorders.

The prediction of ACSL4 ubiquitin E3 ligase, based on Ubibrowser data, suggests the potential involvement of MDM2 in the regulation of ACSL4. To further investigate this effect, we conducted additional experiments. Currently, it has been confirmed that melatonin can modulate the expression of MDM2; however, whether it promotes or inhibits its expression remains a subject of controversy [25–31]. In this study, we provide novel evidence demonstrating that melatonin enhances MDM2 expression to subsequently suppress ACSL4-mediated ferroptosis in an MCAO model. Nevertheless, our findings would greatly benefit from additional clinical samples and cohort data to strengthen their reliability. Hence, this aspect will be a primary focus of our future research endeavors.

In summary, the findings of this study validate the potential of Melatonin in enhancing neuronal protection against ischemic stroke. Further investigation into its mechanism reveals that Melatonin modulates ACSL4 ubiquitination and influences ferroptosis by boosting MDM2 expression, thereby leading to its therapeutic efficacy in treating stroke.

## Supplementary Materials

Supplementary Table 1
