# Supplementary material for: Melatonin improves stroke through MDM2-mediated ubiquitination of ACSL4
Source: Aging (Albany NY). 2024 Jan 29;16(2):1925–37. doi: 10.18632/aging.205469 (PMC10866433; doi:10.18632/aging.205469)
Supplement: Supplementary Table 1 [file aging-16-205469-s001.pdf]

SUPPLEMENTARY TABLE

Supplementary Table 1. The primer sequences of qRT-PCR.

Gene symbol	Forward sequence (5′–3′)	Reverse sequence (5′–3′)
ACSL4	ACTGGCGATATTGGAGAAT	CACATAGGACTGGTCACTT
MDM2	GGCGAGCTTGGCTGCTTC	TGAGTCCGATGATTCCTGCTG
β-actin	AGATCAAGATCATTGCTCCTCCT	ACGCAGCTCAGTAACAGTCC
